# Comparison of clinical features and cerebral glucose metabolism between patients with major depressive disorder and Parkinson’s with depression

**DOI:** 10.1186/s12888-025-07750-y

**Published:** 2026-01-03

**Authors:** Meichen Liu, Xueting Xie, Yudan Liu, Hongbo Feng, Xinyao Wang, Xuemei Du, Huimin Zhang

**Affiliations:** 1 Department of Neurology, The First Affiliated Hospital, Dalian Medical University, Dalian, China; 2 Department of Nuclear Medicine, The First Affiliated Hospital, Dalian Medical University, Dalian, China

**Keywords:** Parkinson’s disease, Major depressive disorder, Cognitive, Clinical features, ^18^F-FDG, Positron emission tomography

## Abstract

**Background:**

Whether there are differences in clinical symptoms and brain glucose metabolism between major depressive disorder (MDD) and Parkinson’s comorbid depression (DPD) is unclear. The aims of this study were to investigate the differences in characteristics in clinical and glucose metabolism between MDD and DPD and their internal correlation.

**Methods:**

We enrolled 30 MDD patients, 25 DPD patients, and 20 Parkinson’s patients without depression (PD-ND), all of whom were evaluated by neuropsychological scales and position emission tomography (PET) imaging. We compared the differences in depressive symptoms, cognitive symptoms, and glucose metabolism imaging and analyzed metabolic characteristics and their correlation with depression.

**Results:**

Compared with MDD, DPD has relatively mild depressive symptoms and is dominated by general systemic symptoms. Meanwhile, the cognitive impairment of DPD is more severe, mainly in executive function. Furthermore, both DPD and MDD have extended metabolism changes in the frontal and parietal lobes and the limbic system. The FDG metabolism in the left posterior medial temporal lobe was significantly lower in patients with DPD compared to those with MDD, and this reduction was positively correlated with the severity of depressive symptoms.

**Conclusions:**

MDD and DPD have some brain regions with common trends, suggesting that both of them may share a common pathophysiological basis. The specific changes, such as hypometabolism in the left primary visual cortex and right inferior lateral temporal lobe, and the hypermetabolism of the right lentiform nucleus, may contribute to the early differentiation of DPD or MDD in patients with depression.

**Clinical trial number:**

ChiCTR2400092675 (Date of Registration: 2024-11-21).

**Supplementary Information:**

The online version contains supplementary material available at 10.1186/s12888-025-07750-y.

## Introduction

Depression in Parkinson’s disease (DPD) has emerged as a prevalent and significant complication that cannot be ignored. Numerous studies have demonstrated that a significant number of Parkinson’s disease (PD) patients exhibit depressive symptoms in the early stage of their disease progression [1, 2]. These symptoms not only accelerate the progression of the disease but also dramatically diminish the patient’s quality of life, contributing to a key factor of disability [3, 4]. Yoon and colleagues further revealed that a history of depression heightens the risk of developing PD by a factor of 3.2 times [5]. In the diagnostic criteria for the prodromal stage of PD, depression has been clearly identified as a risk factor for the onset of PD [6]. However, a considerable proportion of patients with Parkinson’s with depression have not been treated accurately, which is related to the potential mechanism of DPD not having been elucidated [7]. Consequently, it is of great significance to identify specific clinical features in major depressive disorder (MDD) patients or to identify biomarkers that can predict the future development of PD.

It is currently believed that psychiatric symptoms such as depression in PD often antecede motor symptoms. This indicates that these symptoms are not merely a consequence of disease burden or physical limitations; rather, they may represent early indicators of disease progression [8]. This challenges the diagnostic approach to PD when it presents with depressive symptoms. In recent years, neuroimaging biomarkers have emerged as a means to address this gap by identifying disease-specific patterns prior to the emergence of motor symptoms. Integrating non-motor features with imaging studies could offer a strategy for the early detection of PD [9]. ^18^F-fluorodeoxyglucose (^18^F-FDG) positron emission tomography (PET) has been employed to evaluate brain glucose metabolism, which reflects neuronal activity. A study by Huang and colleagues revealed that, compared to controls, DPD exhibited increased metabolism in the amygdala [10]. Furthermore, Prange et al. discovered that early alterations in the structure of the limbic system were correlated with depressive symptoms in PD [11].

In MDD, Vazquez et al. identified hypometabolism in subcortical regions associated with the limbic system, including the entorhinal, orbitofrontal, and cingulate cortices, as well as the hippocampus, amygdala, thalamus, and striatum in animal models [12]. Another meta-analysis showed significantly reduced glucose metabolism in the bilateral insula and left putamen, as well as the right caudate nucleus and cingulate gyrus in MDD patients, with a notable increase in glucose metabolism in the right thalamic pulvinar and anterior vermis [13]. While current studies indicate abnormal glucose metabolism in both DPD and MDD, the findings are not consistent, and few studies have conducted a comparative analysis of the neuroimaging features between DPD and MDD. In addition, both MDD and DPD are characterized by depressive symptoms, but the former is more closely associated with abnormalities in the prefrontal-limbic system loop [14, 15]. The occurrence of DPD may be superimposed on PD-specific dopaminergic neuron degeneration, neuroinflammation, etc [16]. Therefore, it is hypothesized that there could be specific differences in brain metabolic patterns between the two, which may be used as potential biomarkers to differentiate between MDD and DPD.

Differentiating between DPD and MDD in clinical settings can be particularly challenging, especially during the early phases of PD, and there is currently a lack of objective biomarker-assisted judgment. We hypothesis that DPD and MDD may share certain characteristics of glucose metabolism but also exhibit distinct differences. To investigate this, we enrolled three groups: MDD, DPD, and Parkinson’s patients without depression (PD-ND) to examine the heterogeneity of depressive symptoms and the variations in glucose metabolism between the MDD group and DPD groups, as well as between the DPD and PD-ND groups. Additionally, we aim to explore the correlation between depressive symptoms and specific brain regions in both the MDD and DPD groups. The aim is to provide new insights into the differential diagnosis of depression in patients with Parkinson’s disease.

## Subjects and methods

### participants

**Fig. 1:**
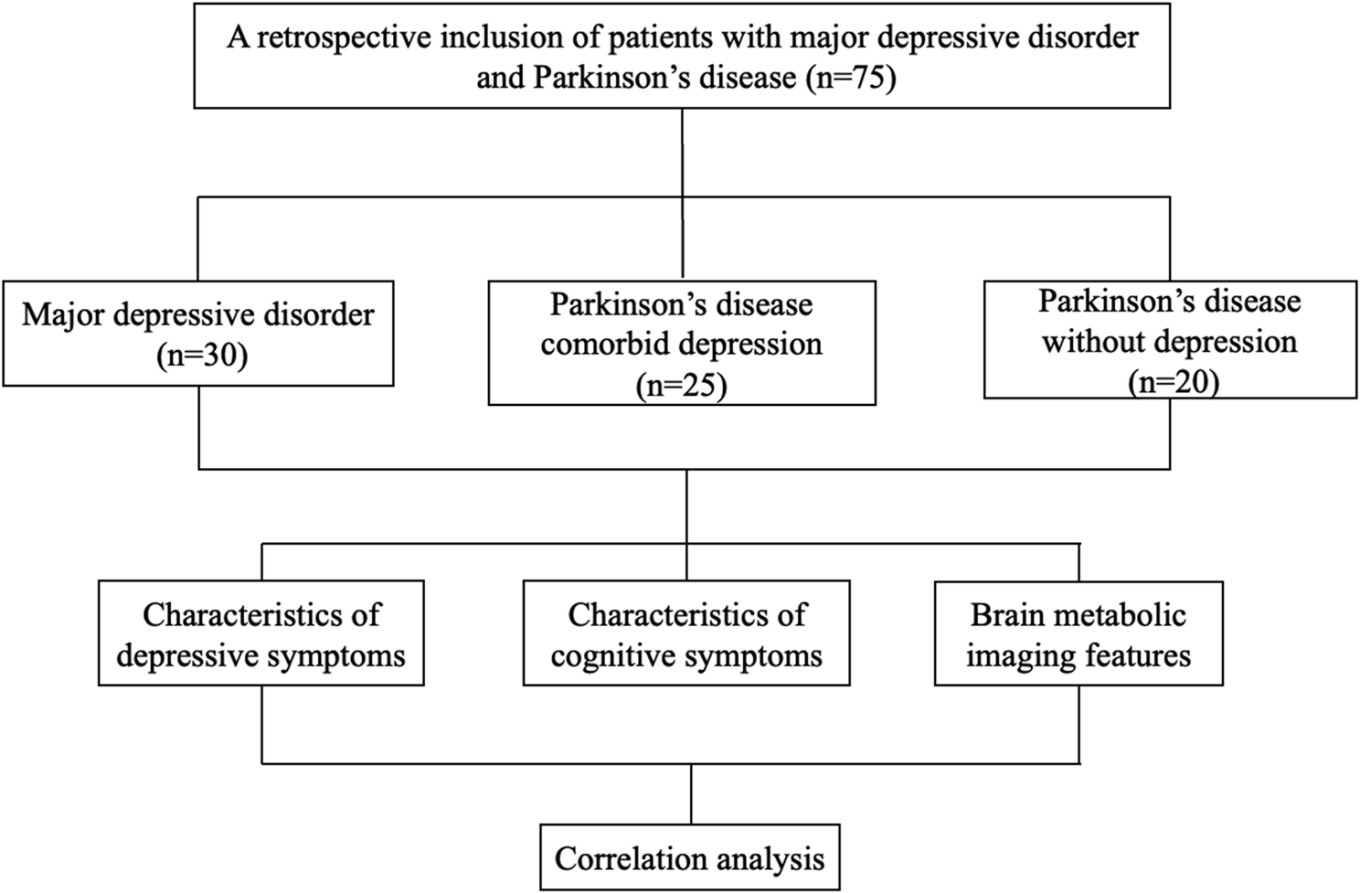
Flow chart

Seventy-five patients were admitted to the outpatient clinic and wards of the Department of Neurology at the First Affiliated Hospital of Dalian Medical University from September 2013 to July 2023. 30 MDD, 25 DPD, and 20 PD-ND were included. All participants underwent ^18^F-FDG PET at the PET center (Fig. 1). All participants were right-handed and had a score on the Chinese Mini Mental State Examination scale (CMMS) ≥ 24. All participants with major depressive disorder were clinically assessed and diagnosed by a psychiatrist according to DSM-V criteria [17]. All PD patients meet the diagnostic criteria of Parkinson’s disease and have a clinical diagnosis of idiopathic PD confirmed by a neurologist [18]. Patients who met the DSM-V depression diagnostic criteria and had Hamilton Depression Scale (HAMD) ≥ 8 points were included in the DPD group. Patients who met the PD diagnostic criteria but did not meet the DSM-V depression diagnostic criteria and had HAMD < 8 were included in the PD-ND group.

The exclusion criteria for all groups are ① patients with other diagnostic diseases in line with DSM-V except anxiety and depression; ② other neurological diseases, such as dementia, epilepsy, brain tumors, and cerebrovascular diseases; ③ A history of brain trauma with neurological sequelae; ④ suffering from hyperthyroidism, hypothyroidism, and other metabolic diseases or rheumatic immune diseases; ⑤ serious heart, lung, liver, kidney, and brain diseases, such as cancer; ⑥ Have a history of drug abuse and alcohol abuse; ⑦ pregnancy or lactation; ⑧ uncooperative with PET examination and scale evaluation. The exclusion criteria for the PD group add an additional one: the presence of cerebrovascular disease, drugs, metabolism, tumor, encephalitis, brain trauma caused by Parkinson’s syndrome, or Parkinson’s syndrome. This study was approved by the local Ethics Committee (PJ-KS-KY-2022-319). All of the study participants provided informed consent prior to their participation.

### Data collection and assessment

Demographic information such as age, gender, marital status, and years of education of the subjects was collected. Collect clinical information such as the duration of disease, Montreal Cognitive Assessment (MOCA) scale [19], CMMS scale [20, 21], Hamilton Anxiety Scale (HAMA), HAMD [22], and other clinical information and cerebral glucose metabolism imaging from all PD and MDD patients. In addition, PD patients were evaluated by Hoehn-Yahr staging (H-Y staging) [23] and Unified Parkinson’s Disease Rating Scale-III (UPDRS-III) [19].

### ^18^F-FDG PET imaging

The PET imaging device used in this research was a Siemens Biograph 64, Germany, and the tracer was ^18^F-FDG, which was automatically synthesized by the ^18^F-FDG drug synthesis system Explora FDG4 module using a Siemens Eclipse RD cyclotron with a radiochemical purity > 95%.

All subjects were forbidden to smoke, drink coffee, and eat stability one day before the examination and ate lightly. All anti-Parkinson drugs, antidepressants, and antipsychotics were discontinued 12 h before the examination; all subjects fasted for at least 6 h, rested for 30 min in the morning, and were given an intravenous injection of ^18^F-FDG at a dose of 0.15 mCi/kg. Images were collected after lying flat in a quiet, dark room for 40 min. The PET scans were performed simultaneously by two PET technologists who had been performing PET examinations for more than 5 years and whose grouping of subjects was unknown. First, a CT scan was performed with a tube voltage of 120 kV, a current of 300 mAs, a pitch of 0.8, a reconstruction layer thickness of 3 mm, and a reconstruction matrix of 512 × 512. Then a bed of PET acquisition was performed for 10 min. CT images were selected for attenuation correction. The layer thickness was 3 mm and kept at the same level as CT images. The TrueX reconstruction algorithm is selected for reconstruction. The number of iterations is 3 times, 21 subsets, Gaussian filtering, FWHM is 4 mm, and the matrix is 336 × 336.

### FDG-PET processing and analysis

NeuroQ™, Version 3.8 (Syntermed, Atlanta, USA) was used to analyze the data of all cases. Firstly, the range of the brain image was determined on the sagittal image, and the non-brain tissue was excluded. Then, the radioactive uptake of the scalp was removed by adjusting the background threshold, and only the brain tissue image was retained. After the case image was aligned to the standard template by rigid body registration, it was transformed to the system standard template to obtain the brain metabolism of each brain region (a total of 47 brain regions) and the difference with the built-in database of the system. The normal controls (NC) have no neuropsychiatric diseases, which comes from the healthy control group of the built-in database of Neuro-Q software. Relevant articles have been published, and quantitative analysis of Neuro-Q software and other methods has also been carried out to prove the reliability. The software has been applied to the analysis of epilepsy and different neurodegenerative diseases [24–26]. We use the average value between all pixels in the entire brain as a standardized reference.

### Statistical analysis

SPSS 27.0 statistical software (IBM, New York, United States) was used for statistical analysis of all data. The measurement data conforming to the normal distribution were expressed as mean ± standard deviation (x ± s). The independent sample t-test was used for comparison between the two groups, and one-way analysis of variance was used for comparison between multiple groups. The measurement data that did not conform to the normal distribution were expressed as median (quartile) [M (P_25_, P_75_)]. The non-parametric test was used for comparison between groups. The Mann-Whitney U test was used for comparison between two groups, and the Kruskal-Wallis H test was used for comparison between multiple groups. The count data were expressed as a rate, and the chi-square test or Fisher’s exact probability method was used for comparison between groups. Pearson or Spearman correlation analysis was used for correlation analysis. *P* < 0.05 indicated that the difference was statistically significant.

## Results

### Patient characteristics

The basic characteristics and neuropsychological measures of the MDD, DPD, and PD-ND groups are shown in (Table 1). The mean ages of these groups were 56.17 (SD = 10.10), 60.36 (SD = 8.90), and 59.95 (SD = 7.94), respectively. The MDD group had higher total scores in the HAMD compared to the DPD group. Except for the general somatic symptoms, the MDD had higher scores in other component parts of HAMD compared with the DPD group (Table 2). The MoCA total score, visual space and execution ability, naming ability, attention, language ability, and orientation score of the DPD group were lower than those of the MDD group (*p* < 0.05). In addition, the visual space and executive ability scores of the DPD group were lower than those of the PD-ND group, and the difference was statistically significant (*p* < 0.05).

**Table 1:** Basic characteristics of MDD, DPD, and PD-ND groups

	MDD (*n* = 30)	DPD (*n* = 25)	PD-ND (*n* = 20)	Overall	MDD vs. DPD	DPD vs. PD-ND	MDD vs. PD-ND
				*p*-value	*p*-value	*p*-value	*p*-value
Age(year)	56.17 ± 10.10	60.36 ± 8.90	59.95 ± 7.94	0.184^a^	0.108^e^	0.874^e^	0.166^e^
**Gender**							
Female	22(73.3%)	11(44.0%)	10(50.0%)	0.067^b^	0.053^f^	0.686^b^	0.092^b^
Male	8(26.7%)	14(56.0%)	10(50.0%)				
**Marital status**							
Wedlock	29(96.7%)	21(84.0%)	17(85.0%)	0.432^b^	0.197^g^	0.836^b^	0.289^g^
Dissociation	0(0.0%)	2(8.0%)	1(5.0%)				
Be widowed	1(3.3%)	2(8.0%)	2(10.0%)				
Education(year)	9.00(8.75, 12.00)	12.00(9.00, 14.00)	11.50(9.00, 15.00)	0.241^c^	0.059^d^	0.704^d^	0.17^d^
Duration(year)	3.63(0.56, 8.56)	3.00(2.75, 5.50)	2.50(2.00, 4.50)	0.552^c^	0.186^d^	0.139^d^	0.042^d*^
H&Y stage(off)	-	1.75(1.00, 2.00)	2.00(1.50, 2.50)	-	-	0.092^d^	-
UPDRS-III(off)	-	20.00(14.25, 28.75)	25.00(19.00, 38.00)	-	-	0.188^d^	-

Note: Data expressed as mean ± standard deviation, n(n%) and median (quartile). a, age data analyzed with single factor analysis of variance; b, gender and marital status data analyzed with Chi-square test. c, education and duration data analyzed with kruskal-wallis test; d, H&Y stage and UPDRS-III data analyzed with Mann-Whitney u test; e, age data analyzed with two sample t test, f, gender status data analyzed with Chi-square test and Yates adjusted; g, marital status data analyzed with Fisher exact probability. Abbreviations: MDD = Major depressive disorder group, DPD = Parkinson’s disease comorbid depression group, PD-ND = Parkinson’s disease group without depression, H&Y, Hoehn and Yahr scale score, UPDRS-III, The motor part of Unified Parkinson’s Disease Rating Scale. *p < 0.05

**Table 2:** Comparison of depressive symptoms and cognitive symptoms among the three groups

	MDD	DPD	PD-ND	MDD vs. DPD	DPD vs. PD-ND	MDD vs. PD-ND
				*p*-value	*p*-value	*p*-value
**HAMA**	26.90 ± 6.50	16.60 ± 4.54	9.70 ± 3.51	<0.001^a***^	<0.001^a***^	<0.001^a***^
**HAMD-17**	20.40 ± 4.44	15.88 ± 3.62	6.00 ± 0.80	<0.001^a***^	<0.001^a***^	<0.001^a***^
Anxiety/ Somatization	6.53 ± 1.80	5.44 ± 1.36	2.5(2.0,3.0)	0.015^a*^	<0.001^b***^	<0.001^b***^
Weight	1.00(0.00, 2.00)	1.00(0.00, 2.00)	0(0.0,0.0)	0.956^b^	0.016^b*^	0.030^b*^
Cognitive impairment	3.00(2.00, 5.00)	2.00(1.00, 3.00)	1.0(1.0,1.0)	0.010^b*^	0.023^b*^	<0.001^b***^
Retardation	4.00(4.00, 5.25)	3.00(2.00, 4.00)	0(0.0,0.0)	0.003^b**^	<0.001^b***^	<0.001^b***^
Sleep disturbance	4.00(2.00, 6.00)	3.00(1.00, 4.00)	1.0(1.0,1.0)	0.019^b*^	<0.001^b***^	<0.001^b***^
General somatic symptoms	1.00(1.00, 2.00)	2.00(1.50, 2.00)	1.0(1.0,1.0)	0.004^b**^	<0.001^b***^	0.009^b**^
**CMMS**	28.00(27.00, 29.25)	26.00(25.00, 27.00)	26.00(25.00, 27.00)	<0.001^b***^	0.584^b^	<0.001^b***^
**MoCA**	25.20 ± 2.55	21.72 ± 3.45	23.20 ± 3.87	<0.001^a***^	0.183^a^	0.150^a^
executive/ visuospatial	4.00(3.00, 5.00)	3.00(3.00, 4.00)	4.00(3.25, 4.75)	0.001^b**^	0.003^b**^	0.890^b^
naming	3.00(3.00, 3.00)	3.00(2.00, 3.00)	3.00(3.00, 3.00)	0.005^b**^	0.371^b^	0.490^b^
attention	2.00(0.00, 3.00)	0.00(0.00, 3.00)	2.00(0.00, 4.00)	0.280^b^	0.214^b^	0.930^b^
language	6.00(5.00, 6.00)	5.00(4.00, 6.00)	5.00(4.25, 6.00)	0.002^b**^	0.549^b^	0.003^b**^
abstraction	2.00(2.00, 3.00)	2.00(1.00, 2.00)	2.00(1.25, 3.00)	0.011^b*^	0.383^b^	0.180^b^
delay memory	2.00(1.00, 2.00)	2.00(0.00, 2.00)	2.00(1.00, 2.00)	0.272^b^	0.665^b^	0.680^b^
orientation	6.00(6.00, 6.00)	6.00(5.00, 6.00)	6.00(5.00, 6.00)	0.005^b**^	0.918^b^	0.100^b^

Note: Data expressed as mean ± standard and median (quartile). a, data analyzed with two sample t test, b, data analyzed with Mann-Whitney u test; Abbreviations: MDD = Major depressive disorder group; DPD = Parkinson’s disease comorbid depression; PD-ND = Parkinson’s disease without depression; CMMS, Chinese Mini Mental State Examination scale; MOCA, Montreal Cognitive Assessment; HAMA, Hamilton Anxiety Scale; HAMD, Hamilton Depression Scale. *p < 0.05, **p < 0.01, ***p < 0.001

### The cerebral metabolism among the three groups

Compared with NC, there is significant hypometabolism in the bilateral medial frontal cortex, bilateral inferior parietal cortex, left anterior cingulate cortex, left Broca’s region, left posterior cingulate cortex, bilateral inferior frontal cortex, and left inferior lateral anterior temporal cortex in both MDD and DPD (*p* < 0.05) (FDR *p* < 0.05). In contrast, there is significant hypermetabolism in the left associative visual cortex, right thalamus, left lentiform nucleus, and bilateral posterior medial temporal cortex in both MDD and DPD (*p* < 0.05) (FDR *p* < 0.05) (Table 3).

**Table 3:** Anomalous areas of cerebral glucose metabolism among MDD, DPD and NC

Brain regions	MDD	DPD	NC	MDD vs. NC				DPD vs. NC				MDD vs. DPD			
	(*n* = 30)	(*n* = 25)	(*n* = 50)	*p*-value	adjusted *p*-value	Cohen’d	95%CI	*p*-value	Adjusted *p*-value	Cohen’d	95%CI	*p*-value	adjusted *p*-value	Cohen’d	95%CI
**Metabolic decreases in both MDD and DPD**															
Right medial frontal cortex	1.04 ± 0.04	1.04 ± 0.04	1.07 ± 0.04	0.008^**^	0.021^#^	-0.640	[-1.110,-0.180]	0.013^*^	0.033^#^	-0.621	[-1.111,-0.131]	0.837	0.913	-0.075	[-0.606,0.456]
Left medial frontal cortex	1.02 ± 0.06	1.02 ± 0.05	1.06 ± 0.03	0.004^**^	0.011^#^	-0.780	[-1.240,-0.310]	0.002^**^	0.007^##^	-0.952	[-1.456,-0.448]	0.888	0.927	0.053	[-0.478,0.584]
Right inferior parietal cortex	1.02 ± 0.05	1.03 ± 0.04	1.07 ± 0.03	< 0.001^***^	< 0.001^###^	-1.130	[-1.620,-0.650]	< 0.001^***^	< 0.001^###^	-1.086	[-1.597,-0.575]	0.635	0.759	-0.143	[-0.687,0.401]
Left inferior parietal cortex	1.01 ± 0.05	1.01 ± 0.05	1.06 ± 0.04	< 0.001^***^	< 0.001^###^	-1.090	[-1.570,-0.600]	< 0.001^***^	< 0.001^###^	-1.110	[-1.622,-0.598]	0.931	0.935	-0.021	[-0.561,0.519]
Left anterior cingulate cortex	1.07 ± 0.05	1.08 ± 0.05	1.12 ± 0.04	< 0.001^***^	< 0.001^###^	-1.170	[-1.660,-0.680]	< 0.001^***^	< 0.001^###^	-1.120	[-1.633,-0.607]	0.667	0.784	-0.124	[-0.668,0.420]
Left Broca’s region	1.13 ± 0.03	1.14 ± 0.03	1.20 ± 0.03	< 0.001^***^	< 0.001^###^	-2.300	[-2.880,-1.720]	< 0.001^***^	< 0.001^###^	-1.822	[-2.384,-1.260]	0.348	0.476	-0.321	[-0.886,0.244]
Left posterior cingulate cortex	1.24 ± 0.03	1.25 ± 0.04	1.27 ± 0.04	< 0.001^***^	< 0.001^###^	-0.860	[-1.330,-0.380]	0.007^**^	0.019^#^	-0.689	[-1.182,-0.196]	0.558	0.693	-0.150	[-0.694,0.394]
Left inferior frontal cortex	1.07 ± 0.04	1.06 ± 0.03	1.12 ± 0.04	< 0.001^***^	< 0.001^###^	-1.270	[-1.760,-0.770]	< 0.001^***^	< 0.001^###^	-1.698	[-2.250,-1.146]	0.317	0.443	0.268	[-0.276,0.812]
Right inferior frontal cortex	1.10 ± 0.04	1.09 ± 0.03	1.16 ± 0.03	< 0.001^***^	< 0.001^###^	-1.748	[-2.280,-1.220]	< 0.001^***^	< 0.001^###^	-2.101	[-2.687,-1.515]	0.367	0.488	0.255	[-0.289,0.799]
Left inferior lateral anterior temporal cortex	0.89 ± 0.02	0.88 ± 0.03	0.93 ± 0.05	< 0.001^***^	< 0.001^###^	-1.010	[-1.490,-0.530]	< 0.001^***^	< 0.001^###^	-1.112	[-1.625,-0.599]	0.352	0.477	0.255	[-0.289,0.799]
**Metabolic increases in both MDD and DPD**															
Left associative visual cortex	1.08 ± 0.03	1.06 ± 0.03	1.05 ± 0.02	< 0.001^***^	< 0.001^###^	1.390	[0.890,1.890]	0.014^*^	0.035^#^	0.745	[0.250,1.240]	0.055	0.114	0.553	[0.005,1.101]
Right Thalamus	1.08 ± 0.05	1.07 ± 0.04	1.01 ± 0.08	< 0.001^***^	< 0.001^###^	1.040	[0.560,1.520]	< 0.001^***^	< 0.001^###^	0.795	[0.298,1.292]	0.199	0.305	0.362	[-0.182,0.906]
Left lentiform Nucleus	1.25 ± 0.05	1.25 ± 0.07	1.20 ± 0.09	0.001^**^	0.001^##^	0.700	[0.230,1.160]	0.016^*^	0.039^#^	0.609	[0.119,1.099]	0.812	0.898	0.061	[-0.551,0.673]
Left posterior medial temporal cortex	0.93 ± 0.03	0.91 ± 0.02	0.90 ± 0.03	< 0.001^***^	< 0.001^###^	1.290	[0.790,1.780]	0.003^**^	0.009^##^	0.699	[0.206,1.192]	0.010^*^	0.026^#^	0.705	[0.151,1.259]
Right posterior medial temporal cortex	0.90 ± 0.03	0.88 ± 0.02	0.86 ± 0.02	< 0.001^***^	< 0.001^###^	1.440	[0.930,1.940]	< 0.001^***^	< 0.001^###^	0.967	[0.462,1.472]	0.031^*^	0.067	0.581	[0.032,1.130]
Pons	0.68 ± 0.03	0.67 ± 0.02	0.64 ± 0.02	< 0.001^***^	< 0.001^###^	1.700	[1.170,2.220]	< 0.001^***^	< 0.001^###^	1.219	[0.700,1.738]	0.065	0.127	0.542	[-0.007,1.091]
Right cerebellum	0.91 ± 0.04	0.92 ± 0.04	0.89 ± 0.04	0.004^**^	0.011^#^	0.680	[0.220,1.150]	0.001^**^	0.001^##^	0.853	[0.353,1.353]	0.575	0.693	-0.150	[-0.694,0.394]
Vermis	0.95 ± 0.05	0.96 ± 0.04	0.90 ± 0.04	< 0.001^***^	< 0.001^###^	1.360	[0.860,1.860]	< 0.001^***^	< 0.001^###^	1.420	[0.887,1.953]	0.864	0.923	-0.044	[-0.588,0.500]
Left cerebellum	0.93 ± 0.04	0.93 ± 0.04	0.90 ± 0.03	0.001^**^	0.001^##^	0.880	[0.410,1.350]	< 0.001^***^	< 0.001^###^	1.100	[0.588,1.612]	0.573	0.693	-0.176	[-0.720,0.368]
**Metabolic decreases only in MDD**															
Left sensorimotor cortex	1.04 ± 0.02	1.07 ± 0.03	1.06 ± 0.04	0.003^**^	0.009^##^	-0.630	[-1.090,-0.170]	0.444	0.574	0.187	[-0.294,0.668]	< 0.0001^***^	< 0.0001^###^	-1.178	[-1.754,-0.602]
Right superior parietal cortex	0.93 ± 0.05	0.97 ± 0.04	0.97 ± 0.05	0.001^**^	0.001^##^	-0.790	[-1.260,-0.320]	0.852	0.917	-0.047	[-0.527,0.434]	0.003^**^	0.009^##^	-0.828	[-1.359,-0.297]
Right Broca’s region	1.09 ± 0.03	1.11 ± 0.03	1.11 ± 0.03	0.010^*^	0.026^#^	-0.600	[-1.060,-0.130]	0.700	0.816	-0.094	[-0.574,0.387]	0.064	0.127	-0.520	[-1.080,0.040]
**Metabolic increases only in MDD**															
Right associative visual cortex	1.06 ± 0.03	1.06 ± 0.03	1.04 ± 0.02	0.004^**^	0.011^#^	0.670	[0.210,1.130]	0.093	0.162	0.456	[-0.030,0.942]	0.535	0.680	0.171	[-0.373,0.715]
Right posterior cingulate cortex	1.21 ± 0.02	1.21 ± 0.03	1.20 ± 0.03	0.017^*^	0.039^#^	0.550	[0.090,1.010]	0.270	0.397	0.272	[-0.210,0.754]	0.288	0.419	0.306	[-0.255,0.867]
Left caudate nucleus	1.03 ± 0.06	0.94 ± 0.05	0.97 ± 0.07	< 0.001^***^	< 0.001^###^	0.900	[0.430,1.370]	0.078	0.149	-0.392	[-0.876,0.092]	< 0.001^***^	< 0.001^###^	1.580	[0.978,2.182]
Right caudate nucleus	1.04 ± 0.04	0.97 ± 0.05	0.96 ± 0.09	< 0.001^***^	< 0.001^###^	0.900	[0.430,1.380]	0.480	0.615	0.137	[-0.344,0.618]	< 0.001^***^	< 0.001^###^	1.163	[0.592,1.734]
Left Thalamus	1.08 ± 0.05	1.06 ± 0.05	1.04 ± 0.07	0.003^**^	0.009^##^	0.660	[0.200,1.120]	0.060	0.121	0.409	[-0.075,0.893]	0.217	0.329	0.341	[-0.203,0.885]
Midbrain	0.75 ± 0.02	0.73 ± 0.03	0.71 ± 0.04	< 0.001^***^	< 0.001^###^	1.110	[0.620,1.590]	0.035^*^	0.075	0.502	[0.015,0.989]	0.017^*^	0.039^#^	0.717	[0.158,1.276]
**Metabolic decreases only in DPD**															
Left primary visual cortex	1.14 ± 0.08	1.12 ± 0.08	1.17 ± 0.05	0.060	0.121	-0.500	[-0.960,-0.040]	0.004^**^	0.011^#^	-0.868	[-1.368,-0.368]	0.298	0.429	0.288	[-0.254,0.830]
Right inferior lateral anterior temporal cortex	0.89 ± 0.03	0.88 ± 0.02	0.90 ± 0.03	0.575	0.693	-0.130	[-0.580,0.320]	0.017^*^	0.039^#^	-0.498	[-0.985,-0.011]	0.090	0.161	0.471	[-0.073,1.015]
Right inferior lateral posterior temporal cortex	0.99 ± 0.03	0.97 ± 0.03	0.99 ± 0.04	0.935	0.935	0.030	[-0.420,0.480]	0.003^**^	0.009^##^	-0.696	[-1.189,-0.203]	0.001^**^	0.001^##^	0.925	[0.359,1.491]
Left middle frontal cortex	1.11 ± 0.03	1.10 ± 0.03	1.12 ± 0.03	0.027^**^	0.059	-0.510	[-0.970,-0.050]	0.001^**^	0.001^##^	-0.836	[-1.334,-0.338]	0.333	0.460	0.293	[-0.247,0.833]
**Metabolic increases only in DPD**															
Right lentiform nucleus	1.18 ± 0.06	1.22 ± 0.05	1.16 ± 0.07	0.100	0.166	0.370	[-0.080,0.830]	0.001^**^	0.001^##^	0.851	[0.352,1.350]	0.052	0.109	-0.543	[-1.088,0.002]

Notes: *p < 0.05, **p < 0.01, ***p < 0.001; FDR adjusted, #*p* < 0.05, ##*p* < 0.01, ###*p* < 0.001. **Abbreviations**: MDD = Major depressive disorder group, DPD = Parkinson ‘s disease and major depressive disorder group, NC = Normal control group

Compared with the DPD group, the brain regions with unique changes in the MDD group were as follows: the FDG metabolism of the right associative visual cortex, right posterior cingulate cortex, bilateral caudate nucleus, the left thalamus, and midbrain in the MDD group was increased, while the left sensorimotor cortex, right superior parietal lobule, right Broca’s region, and left parietotemporal cortex were reduced (*p* < 0.05) (FDR *p* < 0.05). Compared with the MDD group, the brain regions with unique changes in the DPD group were as follows: the FDG metabolism of the left primary visual cortex, right inferior lateral posterior temporal cortex, right inferior lateral anterior temporal lobe, and left middle frontal cortex in the DPD group decreased, while the FDG metabolism of the right lenticular nucleus increased. Further comparison of the degree of FDG metabolism in brain regions with the same trend in the MDD group and the DPD groups showed that the degree of FDG metabolism in the left posterior medial temporal lobe in the DPD group was lower than that in the MDD group (*p* < 0.05) (FDR *p* < 0.05) (Table 3, Supplement Table 1). There was no significant difference in the degree of FDG metabolism in all brain regions between the DPD group and the PD-ND group (Supplement Table 1).

### Correlated HAMD and brain regions in MDD and DPD

**Fig. 2:**
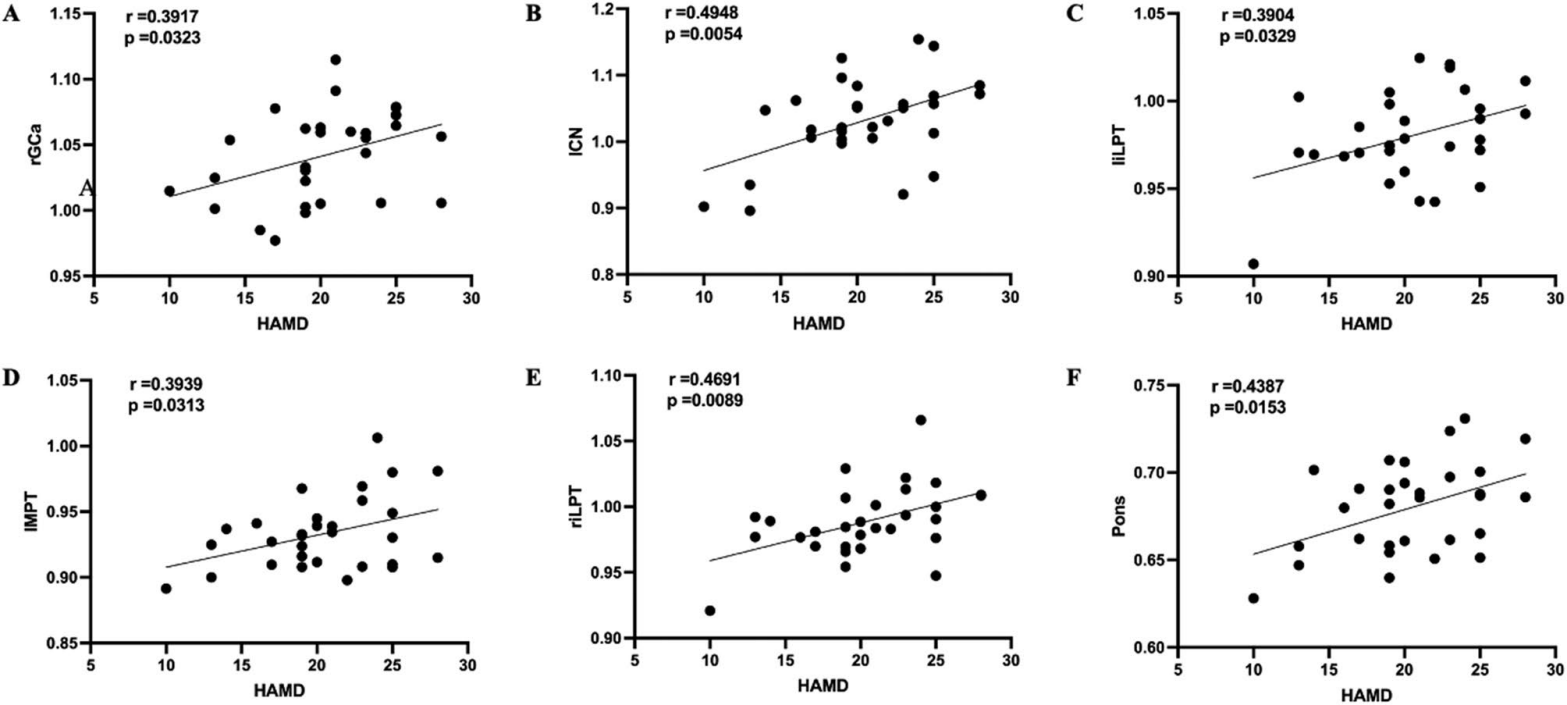
The correlation between the HAMD and FDG metabolism in MDD (**A**, **B**, **C**, **D**, **E**, **F**). Abbreviations: HAMD, Hamilton Depression Scale; FDG, fluorodeoxyglucose; MDD, major depressive disorder; rGCa, right anterior cingulate cortex; lCN, left caudate nucleus; liLPT, left inferior lateral posterior temporal cortex; lMPT, left posterior medial temporal cortex; riLPT, right inferior lateral posterior temporal cortex.

**Fig. 3:**
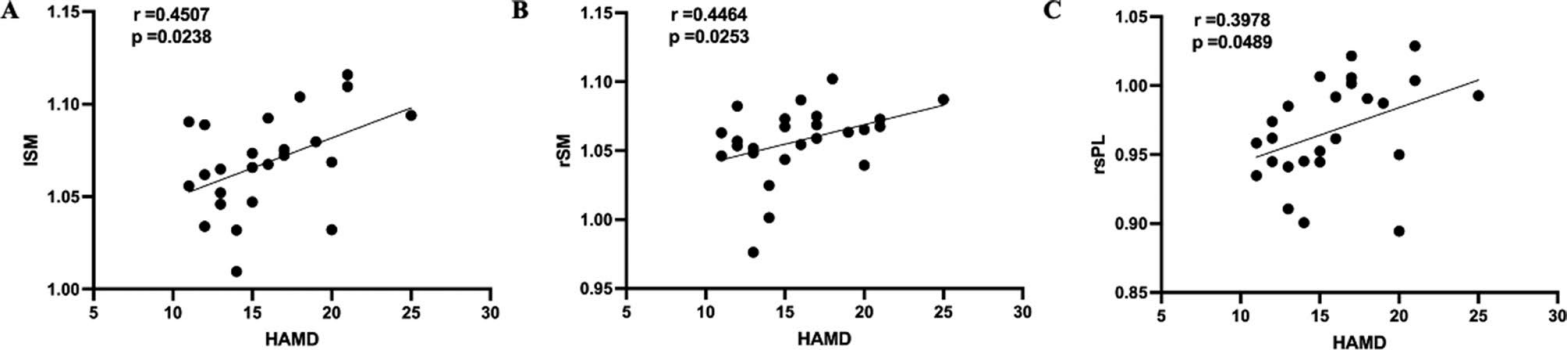
The correlation between the HAMD and FDG metabolism in DPD (**A**, **B**, **C**). Abbreviations: HAMD, Hamilton Depression Scale; FDG, fluorodeoxyglucose; DPD, Parkinson’s disease comorbid depression; lSM, left sensorimotor cortex; rSM, right sensorimotor cortex; rsPL, right superior parietal cortex

The total score of HAMD-17 in the MDD group was positively correlated with the degree of FDG metabolism in the right anterior cingulate cortex (*r* = 0.3917, *p* = 0.0323), left caudate nucleus (*r* = 0.4946, *p* = 0.0054), left inferior lateral posterior temporal cortex (*r* = 0.3904, *p* = 0.0329), left posterior medial temporal cortex (*r* = 0.3939, *p* = 0.0313), right inferior lateral posterior temporal cortex (*r* = 0.4691, *p* = 0.0089), and pons (*r* = 0.4387, *p* = 0.0153) (Fig. 2). The total score of HAMD-17 in the DPD group was only positively correlated with the degree of FDG metabolism in the left sensorimotor cortex (*r* = 0.4507, *p* = 0.0238), right sensorimotor cortex (*r* = 0.4464, *p* = 0.0253), and right superior parietal cortex (*r* = 0.3978, *p* = 0.0489) (Fig. 3).

## Discussion

In this study, neuropsychological scales and ^18^F-FDG PET imaging were used to compare the clinical characteristics and brain metabolic patterns of MDD, DPD, and PD-ND patients, aiming to reveal the neurobiological differences between the two types of depression. The findings revealed that DPD patients exhibited more pronounced systemic symptoms. However, they experienced relatively milder psychological symptoms, including depression, anxiety/somatization, and sleep disturbances, with cognitive deficits primarily affecting visuospatial and executive functions. FDG-PET imaging indicated that both MDD and DPD shared common metabolic abnormalities, notably in the prefrontal cortex and limbic system. Yet, DPD patients displayed unique metabolic characteristics in specific brain regions: hypometabolism in the left visual cortex and hypermetabolism in the right lentiform nucleus, as well as distinct patterns in the temporal lobe and basal ganglia.

Our study shows that DPD patients have more severe systemic symptoms, while MDD patients have more depression, inattention, self-blame, and suicidal tendencies. Although Ahmad et al. posited that there is a significant overlap in symptoms between DPD and MDD, such as low mood and diminished interest [16], contemporary research suggests a distinction where MDD patients suffer from severe depressive symptoms, whereas DPD is characterized by mild to moderate symptomatology [7]. This distinction may be linked to the anhedonia observed in DPD patients, coupled with a lower incidence of self-blame and suicidal tendencies [27, 28]. In addition, physical symptoms such as joint pain and dizziness are common in DPD patients [29, 30], which may be related to more prominent fatigue and pain caused by exercise restriction. The pathological mechanisms of DPD and MDD involve neurotransmitter imbalance, hypothalamic-pituitary-adrenal (HPA) axis dysregulation, and disruptions in gut microbiota. However, the heterogeneity of the diseases, variations among populations, and the tools used for assessment may all influence the outcomes of research studies.

In terms of cognitive function, the scores of visuospatial and executive functions in the DPD group and the PD-ND group were significantly lower than those in the MDD group, and DPD patients showed more extensive cognitive impairment. Meta-analysis showed that the cognitive function of MDD patients was impaired, mainly in executive function, memory, and attention deficits [31, 32], and may deteriorate with the recurrence of the disease [33]. On the contrary, the cognitive impairment of PD patients involves multiple cognitive domains, such as memory, attention, visual space, and executive function, among which executive dysfunction is the most prominent and progressive [34]. Depression is associated with impairment of multiple cognitive domains in PD patients [35, 36]. Longitudinal studies suggest that DPD patients have faster cognitive decline, especially affecting visuospatial, memory, and executive function [37]. This is consistent with our findings that DPD patients have more extensive and severe cognitive impairment, especially in visuospatial and executive function, which may be due to the combined results of depression-related cognitive deficits and PD-related neurodegeneration.

This study reveals that patients with MDD and DPD exhibit distinct metabolic abnormalities in the brain, which is consistent with previous studies on neurotransmitter imbalances and dysfunction in specific brain regions [12, 13, 38]. In line with previous reports, our analysis indicates that MDD patients exhibit elevated metabolic activity in the limbic system, including the posterior cingulate gyrus and thalamus, coupled with reduced metabolism in the sensorimotor cortex. Conversely, DPD patients demonstrate hypermetabolism in the basal ganglia, particularly the lentiform nucleus, alongside a suppression of metabolic activity in the visual cortex. This is corroborated by previous research documenting decreased occipital visual cortex activity in PD patients during visual stimulation tasks [39]. Moreover, PET and functional magnetic resonance imaging (fMRI) studies have previously noted hypermetabolism in the hippocampus of MDD patients [40] and increased activity in the amygdala [41], which corresponds with the metabolic alterations we observed in these brain regions. However, unlike previous studies, our study uniquely and systematically contrasts the metabolic profiles of the two groups of patients within the temporal lobe for the first time. We found that MDD patients displayed significantly higher glucose metabolism in these regions compared to DPD patients (FDR *p* < 0.05). The temporal lobe is an important part of the emotion-related pathway, which suggests that the mechanisms of depression in the two diseases may be different.

Significantly, this study not only confirms the pivotal role of the basal ganglia-thalamic-cortical circuit in DPD [38, 42] but also elucidates the distinct metabolic decline in the right inferior temporal lobe of DPD patients. The functional impairment of this area in processing emotional stimuli has been less extensively documented [43]. In contrast to the research by Ahmad et al. [16] and Wang et al. [44], our findings offer a clearer delineation of the neural network differences between MDD and DPD: MDD is primarily characterized by dysregulation within the emotional regulation circuit, whereas DPD presents with a dual challenge of impaired motion-emotion integration and compromised visual processing networks. These insights furnish novel imaging evidence that enhances our understanding of the neurobiological underpinnings of these two depression subtypes.

Our investigation revealed a significant positive correlation between the severity of depression in MDD patients and FDG metabolism in key brain regions, including the anterior cingulate gyrus, posterior temporal lobe, caudate nucleus, and pons. This result is consistent with the conclusion of the latest study, which posits that excessive activation of amygdalo-pontine connections is associated with depressive symptoms [45]. Although previous research reported an inverse relationship between prefrontal metabolism and depression, our study did not observe this trend [46], which may be attributed to variations in brain region classification methods or statistical analyses employed. In the DPD group, we found that the severity of depressive symptoms positively correlated with metabolic activity in the sensorimotor cortex and the superior parietal lobule. This is congruent with findings from PD-related studies that have identified increased amygdala metabolism in relation to the exacerbation of depression [10, 47]. It is particularly noteworthy that recent clinical studies have shown that transcranial magnetic stimulation intervention in the left motor cortex can enhance the treatment response of MDD patients, indicating a strong association between this region and behavioral activation as well as cognitive function [48]. Based on these findings, the metabolic alterations in the sensorimotor cortex may be the key neural basis for mood regulation disorders in DPD patients. Consequently, targeted interventions in this area could potentially represent a novel therapeutic approach to alleviate depressive symptoms in DPD.

There are some limitations in this study. First, as a single-center cross-sectional investigation, the relatively limited sample size across subgroups may restrict the generalizability of our findings. Second, the use of PET/CT with inherent limitations in CT resolution and the absence of high-resolution structural MRI co-registration prevented partial volume effect (PVE) correction. Moreover, the normal control group was derived from an embedded software database; future studies will incorporate rigorously matched local healthy controls based on demographic characteristics such as age, gender, and ethnicity. Additionally, medication use among participants was not strictly controlled beyond a 12-hour pre-scan withdrawal period. Future work will employ broader inclusion criteria and more comprehensive medication management to better account for potential confounding effects. These refinements will contribute to a more precise characterization of the neurometabolic profiles between MDD and DPD.

## Conclusions

Overall, this study revealed significant differences in depressive symptoms and cognitive function between the DPD and MDD groups. Shared cerebral metabolic patterns suggest potential common pathophysiological mechanisms, while distinct regional alterations in DPD may serve as early biomarkers for differential diagnosis. Future studies integrating multimodal imaging could further elucidate the distinctions between DPD and MDD, providing a more robust scientific basis for early identification.

## Supplementary Information

Below is the link to the electronic supplementary material.

Supplementary Material 1

Supplementary Material 2

Supplementary Material 3

## Data Availability

The publication and supporting materials contain the original contributions made for this work; for additional information, contact the corresponding author.
