# Supplementary material for: Comparison of clinical features and cerebral glucose metabolism between patients with major depressive disorder and Parkinson’s with depression
Source: BMC Psychiatry. 2026 Jan 3;26:110. doi: 10.1186/s12888-025-07750-y (PMC12866246; doi:10.1186/s12888-025-07750-y)
Supplement: Supplementary file 3 — Supplementary Material 3 [file 12888_2025_7750_MOESM3_ESM.docx]

| **Supplement Table 2 The areas of cerebral glucose metabolism between DPD and PD-ND** | | | | | | | | | |
| --- | --- | --- | --- | --- | --- | --- | --- | --- | --- |
| **Brain regions** | **DPD** | **PD-ND** | ***p-value*** | **adjusted *p-value*** | **Brain regions** | **DPD** | **PD-ND** | ***p-value*** | **adjusted *p-value*** |
| Right superior frontal cortex | 1.02±0.04 | 1.03±0.03 | 0.701 | 0.879 | Left caudate nucleus | 0.94±0.05 | 0.97±0.08 | 0.162 | 0.300 |
| Right medial frontal cortex | 1.04±0.04 | 1.04±0.03 | 0.864 | 0.919 | Right caudate nucleus | 0.97±0.05 | 1.00±0.06 | 0.105 | 0.198 |
| Left superior frontal cortex | 0.98±0.02 | 0.98±0.02 | 0.291 | 0.582 | Left inferior frontal cortex | 1.06±0.03 | 1.08±0.02 | 0.092 | 0.096 |
| Left medial frontal cortex | 1.02±0.05 | 1.01±0.06 | 0.725 | 0.835 | Right inferior frontal cortex | 1.09±0.03 | 1.09±0.03 | 0.792 | 0.879 |
| Left sensorimotor cortex | 1.07±0.03 | 1.06±0.02 | 0.485 | 0.668 | Left Thalamus | 1.06±0.05 | 1.07±0.06 | 0.879 | 0.919 |
| Right sensorimotor cortex | 1.06±0.03 | 1.05±0.02 | 0.564 | 0.746 | Right Thalamus | 1.07±0.04 | 1.09±0.06 | 0.214 | 0.368 |
| Left superior parietal cortex | 0.94±0.04 | 0.92±0.04 | 0.173 | 0.326 | Right lentiform nucleus | 1.21±0.05 | 1.22±0.09 | 0.727 | 0.835 |
| Right superior parietal cortex | 0.97±0.04 | 0.95±0.05 | 0.199 | 0.368 | Left lentiform Nucleus | 1.25±0.07 | 1.24±0.10 | 0.746 | 0.835 |
| Right middle frontal cortex | 1.12±0.03 | 1.12±0.03 | 0.669 | 0.835 | Right primary visual cortex | 1.08±0.09 | 1.05±0.05 | 0.211 | 0.368 |
| Left middle frontal cortex | 1.10±0.03 | 1.11±0.03 | 0.336 | 0.582 | Left primary visual cortex | 1.12±0.08 | 1.10±0.06 | 0.447 | 0.668 |
| Right inferior parietal cortex | 1.03±0.04 | 1.02±0.05 | 0.489 | 0.668 | Left inferior lateral anterior temporal cortex | 0.88±0.03 | 0.88±0.02 | 0.575 | 0.746 |
| Left inferior parietal cortex | 1.01±0.05 | 1.01±0.05 | 0.793 | 0.879 | Left anterior medial temporal cortex | 0.73±0.04 | 0.74±0.04 | 0.400 | 0.668 |
| Right anterior cingulate cortex | 1.04±0.03 | 1.04±0.04 | 0.82 | 0.879 | Right inferior lateral anterior temporal cortex | 0.88±0.02 | 0.88±0.03 | 0.932 | 0.966 |
| Left anterior cingulate cortex | 1.08±0.05 | 1.07±0.06 | 0.634 | 0.835 | Right anterior medial temporal cortex | 0.71±0.02 | 0.71±0.04 | 0.491 | 0.668 |
| Left associative visual cortex | 1.06±0.03 | 1.05±0.03 | 0.134 | 0.251 | Midbrain | 0.73±0.03 | 0.74±0.05 | 0.581 | 0.746 |
| Right associative visual cortex | 1.06±0.03 | 1.05±0.04 | 0.485 | 0.668 | Left inferior lateral posterior temporal cortex | 0.97±0.02 | 0.97±0.02 | 0.792 | 0.879 |
| Right Broca's region | 1.11±0.03 | 1.11±0.03 | 0.919 | 0.919 | Left posterior medial temporal cortex | 0.91±0.02 | 0.92±0.03 | 0.603 | 0.746 |
| Left Broca's region | 1.14±0.03 | 1.14±0.04 | 0.586 | 0.746 | Right inferior lateral posterior temporal cortex | 0.97±0.03 | 0.96±0.03 | 0.918 | 0.919 |
| Right posterior cingulate cortex | 1.21±0.03 | 1.21±0.04 | 0.966 | 0.966 | Right posterior medial temporal cortex | 0.88±0.02 | 0.88±0.03 | 0.713 | 0.835 |
| Right parietotemporal cortex | 1.08±0.06 | 1.07±0.07 | 0.473 | 0.668 | Pons | 0.67±0.02 | 0.68±0.05 | 0.292 | 0.582 |
| Left posterior cingulate cortex | 1.25±0.04 | 1.24±0.05 | 0.513 | 0.692 | Right cerebellum | 0.92±0.04 | 0.93±0.05 | 0.479 | 0.668 |
| Left parietotemporal cortex | 1.08±0.06 | 1.07±0.08 | 0.431 | 0.668 | Vermis | 0.96±0.04 | 0.96±0.06 | 0.604 | 0.746 |
| Left superior lateral temporal cortex | 1.10±0.03 | 1.10±0.03 | 0.835 | 0.879 | Left cerebellum | 0.93±0.04 | 0.94±0.05 | 0.499 | 0.692 |
| Right superior lateral temporal cortex | 1.08±0.03 | 1.07±0.03 | 0.501 | 0.692 |  |  |  |  |  |
| ***Notes***: *p<0.05; FDR adjusted, #p<0.05. **Abbreviations**: DPD = Parkinson’s disease comorbid depression group, PD-ND = Parkinson’s disease group without depression. | | | | | | | | | |
